# Association of Severe Hyperoxemia Events and Mortality Among Patients Admitted to a Pediatric Intensive Care Unit

**DOI:** 10.1001/jamanetworkopen.2019.9812

**Published:** 2019-08-21

**Authors:** Sriram Ramgopal, Cameron Dezfulian, Robert W. Hickey, Alicia K. Au, Shekhar Venkataraman, Robert S. B. Clark, Christopher M. Horvat

**Affiliations:** 1 Department of Pediatrics, University of Pittsburgh School of Medicine; UPMC Children's Hospital of Pittsburgh, Pittsburgh, Pennsylvania; 2 Department of Critical Care Medicine, University of Pittsburgh School of Medicine, Pittsburgh, Pennsylvania; 3 Safar Center for Resuscitation Research, University of Pittsburgh School of Medicine, Pittsburgh, Pennsylvania; 4 Health Informatics for Clinical Effectiveness, UPMC Children’s Hospital of Pittsburgh, Pittsburgh, Pennsylvania

## Abstract

**Importance:**

A high Pao_2_, termed hyperoxemia, is postulated to have deleterious health outcomes. To date, the association between hyperoxemia during the ongoing management of critical illness and mortality has been incompletely evaluated in children.

**Objective:**

To examine whether severe hyperoxemia events are associated with mortality among patients admitted to a pediatric intensive care unit (PICU).

**Design, Setting, and Participants:**

A retrospective cohort study was conducted over a 10-year period (January 1, 2009, to December 31, 2018); all 23 719 PICU encounters at a quaternary children’s hospital with a documented arterial blood gas measurement were evaluated.

**Exposures:**

Severe hyperoxemia, defined as Pao_2_ level greater than or equal to 300 mm Hg (40 kPa).

**Main Outcomes and Measures:**

The highest Pao_2_ values during hospitalization were dichotomized according to the definition of severe hyperoxemia and assessed for association with in-hospital mortality using logistic regression models incorporating a calibrated measure of multiple organ dysfunction, extracorporeal life support, and the total number of arterial blood gas measurements obtained during an encounter.

**Results:**

Of 23 719 PICU encounters during the inclusion period, 6250 patients (13 422 [56.6%] boys; mean [SD] age, 7.5 [6.6] years) had at least 1 measured Pao_2_ value. Severe hyperoxemia was independently associated with in-hospital mortality (adjusted odds ratio [aOR], 1.78; 95% CI, 1.36-2.33; *P* < .001). Increasing odds of in-hospital mortality were observed with 1 (aOR, 1.47; 95% CI, 1.05-2.08; *P* = .03), 2 (aOR, 2.01; 95% CI, 1.27-3.18; *P* = .002), and 3 or more (aOR, 2.53; 95% CI, 1.62-3.94; *P* < .001) severely hyperoxemic Pao_2_ values obtained greater than or equal to 3 hours apart from one another compared with encounters without hyperoxemia. A sensitivity analysis examining the hypothetical outcomes of residual confounding indicated that an unmeasured binary confounder with an aOR of 2 would have to be present in 37% of the encounters with severe hyperoxemia and 0% of the remaining cohort to fail to reject the null hypothesis (aOR of severe hyperoxemia, 1.31; 95% CI, 0.99-1.72).

**Conclusions and Relevance:**

Greater numbers of severe hyperoxemia events appeared to be associated with increased mortality in this large, diverse cohort of critically ill children, supporting a possible exposure-response association between severe hyperoxemia and outcome in this population. Although further prospective evaluation appears to be warranted, this study’s findings suggest that guidelines for ongoing management of critically ill children should take into consideration the possible detrimental effects of severe hyperoxemia.

## Key Points

**Question:**

Is severe hyperoxemia (arterial oxygen tension ≥300 mm Hg) associated with mortality among critically ill children?

**Findings:**

In this cohort study of 23 719 intensive care encounters from 2009 to 2018 at a children’s hospital, 6250 patients had at least 1 measured arterial oxygen tension value. After adjusting for covariates, severe hyperoxemia appeared to be independently associated with in-hospital mortality, and a stepwise increase in the adjusted odds of mortality was observed with more episodes of severe hyperoxemia.

**Meaning:**

Severe hyperoxemia appeared to be associated with mortality in a large, single-center cohort of critically ill children; prospective data are needed to assess causality.

## Teaser

This cohort study examines whether severe hyperoxemia events are associated with mortality among children admitted to a pediatric intensive care unit.

## Introduction

Oxygen is the most commonly used therapy in the management of critically ill patients. First described at the end of the 20th century, increasing evidence suggests that overuse of oxygen may be associated with harm among both critically ill children and adults.^1,2,3^ Hyperoxia has been demonstrated to induce alveolar injury^4^ and hyperoxemia has been associated with endothelial dysfunction^5^ and decreased coronary blood flow.^6^ Preclinical studies have indicated that hyperoxemia is associated with vasoconstriction and a corresponding reduction in cardiac output.^7^ Oxygen free-radical–mediated damage has been implicated in inflammatory cascades and apoptosis.^8,9,10^ Several large, observational studies have identified hyperoxemia following cardiac arrest in adults as an independent risk factor for mortality,^11,12,13,14^ although not all studies have supported such an association.^15,16^ A recent meta-analysis of 25 prospective, randomized clinical trials that included 16 037 patients identified an increased relative risk of mortality among patients whose therapy was managed with liberal oxygen use compared with oxygen-conservative strategies.^17^

Critically ill children are frequently exposed to supplemental oxygen for prolonged periods and are at theoretic risk of hyperoxemia-related injury and resulting poor outcomes; however, studies of hyperoxemia in critically ill pediatric patients have produced conflicting results. Several smaller studies examining arterial oxygen tension early following cardiac arrest, each including fewer than 250 children, did not find an association between hyperoxemia and mortality.^18,19,20,21,22^ In contrast, 2 large observational studies identified an association between presenting hyperoxemia and mortality among diagnostically diverse cohorts of critically ill children.^23,24^

It remains unclear whether a causal relationship exists between hyperoxemia and outcome or whether an unidentified confounder mediates these findings, such as an association between hyperoxemia and aggressive resuscitation with high concentrations of supplemental oxygen among severely ill patients. In the present study, our objective was to examine whether severe hyperoxemia during hospitalization among patients admitted to a pediatric intensive care unit (PICU) was associated with mortality. To build on previous studies, we sought to determine the possibility of whether an exposure-response association existed between severe hyperoxemia and mortality in a large, acuity-adjusted series of PICU patients. We hypothesized that severe hyperoxemia would be independently associated with mortality and that an increasing number of severe hyperoxemia events would be associated with increasing risk of death.

## Methods

### Study Setting and Inclusion Criteria

We performed a retrospective cohort study from a quaternary care PICU in western Pennsylvania. The study institution is a level I trauma center and serves a catchment area of approximately 5 million people. A dedicated neonatal intensive care unit provides care for infants less than 44 weeks’ corrected gestational age at the time of admission, and patients with congenital heart disease are cared for in a separate, dedicated cardiac intensive care unit; these groups were not included in the present study. We evaluated all encounters in children admitted to the PICU between January 1, 2009, to December 31, 2018. Each hospitalization was treated as a separate encounter. An illness severity measurement was constructed using data from all PICU encounters. Encounters with a measured PaO_2_ during hospitalization were identified and included in the analysis of hyperoxemia. This study followed the Strengthening the Reporting of Observational Studies in Epidemiology (STROBE) reporting guideline.^25^ Prior to data collection, all aspects of this study were approved by the University of Pittsburgh Institutional Review Board with a waiver of informed consent, owing to deidentified data following collection.

### Data Extraction

For each encounter, the following data were abstracted from an electronic, clinical data warehouse using the business intelligence platform SAP BusinessObjects: demographic data (age and sex), hospitalization data (time of admission and discharge), outcome data (discharge or in-hospital mortality), and Pao_2_ values. In addition, we collected clinical data (mean arterial blood pressure, Glasgow Coma Scale measurement, pupillary reflexes, mechanical ventilation) and laboratory data elements (Paco_2_, lactate level, creatinine level, white blood cell count, platelet count) required for risk adjustment scoring. Because patients receiving extracorporeal life support (ECLS) may have a high Pao_2_ level resulting from a venoarterial circuit, use of ECLS was abstracted for all patients. All data points were time stamped.

### Outcome and Exposure

The outcome of interest was in-hospital mortality. Maximum values of Pao_2_ at any time during PICU hospitalization for each encounter were used in the analyses. On the basis of previously published literature,^14,17,24,26^ we selected 300 mm Hg (40 kPa) as the arterial oxygen tension defining severe hyperoxemia. Duration of exposure to severe hyperoxemia was modeled by identifying encounters with at least 3 Pao_2_ values, each recorded at least 3 hours apart, and grouping these encounters into 4 categories based on the number of severely hyperoxemic Pao_2_ values (0, 1, 2, or ≥3).

### Measuring Severity of Illness

To account for patient illness severity, we constructed a modified version of the Pediatric Logistic Organ Dysfunction-2 (m-PELOD-2) score using structured data harbored by the electronic health record, per previously published methods, excluding the ratio of Pao_2_ to fraction of inspired oxygen (Pao_2_/Fio_2_) values from the m-PELOD-2 calculation for the present analyses.^27,28^ The m-PELOD-2 scores range from 0 to 31, with 0 indicating no organ dysfunction and 31 indicating the greatest amount of organ dysfunction as quantified by the score. For risk assessment, m-PELOD-2 scores were derived using data obtained throughout the entire hospitalization. Calibration of the m-PELOD-2 instrument was performed by randomly selecting 75% of all PICU encounters as a development cohort and assessing performance of the resulting model output among the remaining 25% of all PICU encounters.

### Statistical Analysis

Data were summarized with descriptive statistics, using mean (SD) for parametric data and median (interquartile range [IQR]) for nonparametric data. We assessed predictive validity of the m-PELOD-2 by examining the C statistic as a measure of discrimination and calibration by inspection of observed vs predicted mortality calibration belts and use of the Hosmer-Lemeshow goodness-of-fit test, defining inadequate fit a priori as *P* < .05. The 95% CIs of the C statistics were calculated per the method of Delong using the pROC package, version 1.13.0, in R (R Project for Statistical Computing). Calibration was assessed using the GiViTi package, version 1.3, in R to construct calibration belts and the ResourceSelection package, version 0.3-4, in R to perform the Hosmer-Lemeshow goodness-of-fit test based on deciles of observed to predicted mortality. Acceptable calibration was defined a priori as a *P* > .05 using either approach.

Proportions of observed and predicted mortality were examined in strata of 50–mm Hg (6.7 kPa) Pao_2_ increments. Univariable logistic regression models assessed patient age, m-PELOD-2 score, use of ECLS, the number of Pao_2_ values obtained during the encounter, and severe hyperoxemia with in-hospital mortality as the outcome. A multivariable model was constructed incorporating variables with a univariable *P* < .10. Differences in time intervals between admission time and multiple Pao_2_ values were compared using the Wilcoxon rank sum test and the Kruskal-Wallis test. We conducted a sensitivity analysis to assess for residual confounding in the multivariable model by examining the hypothetical influence of an unmeasured, binary confounder of hyperoxemia on mortality using the obsSens package, version 1.0, in R. The unmeasured confounder was modeled with effect sizes of adjusted odds ratios (aORs) of 2, 3, 4, and 5. The threshold for statistical significance in all analyses was an α level of .05, with 2-tailed *P* values determined. Results are reported as odds ratios (ORs) and aORs with 95% CIs. Analyses were conducted using R, version 3.5.1 (R Project for Statistical Computing).

### Additional Analyses

We performed several post hoc analyses to further evaluate the association between hyperoxemia and mortality identified in our initial analyses. To evaluate whether multiple encounters of the same patient may have confounded the results, we performed 2 sensitivity analyses. First, we again conducted the main analysis constructing logistic regression models with each encounter’s maximum Pao_2_, including only the last available encounter for each child. For the second analysis, we included all encounters and constructed a generalized estimating equations logistic regression model, clustering by patient identifier, to account for correlation between recurrent encounters by the same patient.

To further evaluate thresholds of hyperoxemia associated with mortality, we constructed a receiver operator curve (ROC) using maximum Pao_2_ level for each encounter and identified cut points using the Youden method, misclassification cost term, maximized specificity, and maximized sensitivity and specificity. Sensitivity, specificity, positive and negative predictive value, and positive and negative likelihood ratios were identified at each cut point. Univariable and multivariable logistic regression models examined maximum Pao_2_ levels in bins of no hyperoxemia (<300 mm Hg [40 kPa]), severe hyperoxemia (300-499 mm Hg [40- 66.5 kPa]), and extreme hyperoxemia (≥500 mm Hg [66.5 kPa]). To better account for nonparametric predictors, a multivariate adaptive regression splines (MARS) model was constructed using all covariates from the primary analysis.^29^ The MARS model provides added flexibility compared with linear models, such as logistic regression, by incorporating nonlinear relationships of the included variables. We performed 10-fold cross-validation to identify the optimal combination of interaction effects and terms and ranked the terms in order of importance on the basis of reduction of generalized cross-validation estimates. This analysis was performed using the earth, version 5.1.1; caret, version 6.0-8.1; and vip, version 0.1.2; packages of R software. The area under the receiver operating curve (AUROC) of the MARS model was compared with the logistic regression model of the primary analysis using the method of DeLong and the pROC package, version 1.15.0.

To further evaluate a gradient-response association between severe hyperoxemia and mortality, we additionally modeled arterial oxygen tension as an area under the curve (AUC). We selected the subset of patients for whom there were at least 3 daily measurements of Pao_2_ over the initial 3 days following arterial line placement and calculated an AUC using trapezoidal integration. We additionally explored whether paired oxygen saturation as measured by pulse oximetry (Spo_2_) and Fio_2_ values between Pao_2_ measurements could serve as a reliable surrogate of arterial oxygen tension. This testing was accomplished by examining distributions of Pao_2_ measurements obtained within 20 minutes of a documented Spo_2_ value of 100% and a Fio_2_ value. A MARS model was constructed that incorporated the AUC as a continuous variable, adjusting for patient age, m-PELOD-2 score, use of ECLS, and the number of Pao_2_ values obtained during the encounter.

## Results

There were 23 719 PICU encounters in the 10-year study period; 491 children (2.1%) died during hospitalization. There were 174 160 Pao_2_ values measured in 6250 encounters. Among patients with a Pao_2_ value available, 13 422 were male (56.6%), the mean (SD) age was 7.5 (6.6) years, and 405 children (6.5%) died during hospitalization. In-hospital mortality among patients without a measured Pao_2_ value was 0.49%. Demographic data for included encounters are provided in Table 1.

**Table 1.** Patient Demographics

Characteristic	All PICU Encounters	Encounters With Pao_2_ Values (n = 6250)	
		No Hyperoxemia (<300 mm Hg)	Hyperoxemia (≥300 mm Hg)
No. (%)	23 719	4559 (72.9)	1691 (27.1)
Pao_2_, median (IQR), mm Hg	104 (77-145)	97 (75-132)	112 (80-160)
In-hospital mortality, No. (%)	491 (2.1)	162 (3.6)	243 (14.4)
Male, No. (%)	13 422 (56.6)	2531 (55.5)	969 (57.3)
Age, No. (%)			
≤30 d	386 (1.6)	62 (1.4)	39 (2.3)
>30 d to <1 y	3886 (16.4)	730 (16.0)	291 (17.2)
1 to <2 y	2720 (11.5)	470 (10.3)	160 (9.5)
2 to <6 y	5362 (22.6)	818 (17.9)	305 (18.0)
6 to <12 y	4521 (19.1)	948 (20.8)	315 (18.6)
12 to <18 y	5229 (22.0)	1163 (25.5)	443 (26.2)
≥18 y	1615 (6.8)	368 (8.1)	138 (8.2)
Race/ethnicity, No. (%)			
White	17 974 (75.8)	3584 (78.6)	1296 (76.6)
Black	4138 (17.4)	654 (14.3)	247 (14.6)
Other or not stated	1607 (6.8)	321 (7.0)	148 (8.8)
Use of ECLS, No. (%)	146 (0.6)	38 (0.8)	99 (5.9)
Estimated mortality by m-PELOD-2 score, %			
Median (IQR)	0.3 (0.2-0.7)	0.7 (0.3-1.9)	1.8 (0.6-10.2)
Mean (SD)	2.1 (8.4)	3.9 (11.1)	11.8 (21.2)

Abbreviations: ECLS, extracorporeal life support; IQR, interquartile range; m-PELOD 2, modified Pediatric Logistic Organ Dysfunction-2; PICU, pediatric intensive care unit.

The m-PELOD-2 AUROC in the development cohort (n = 17 816) was 0.93 (95% CI, 0.91-0.94) and did not demonstrate acceptable calibration through assessment of the calibration belt and the Hosmer-Lemeshow goodness-of-fit test (both *P* < .001). After recalibration, the AUROC was 0.94 (95% CI, 0.92-0.95) and the goodness-of-fit test indicated acceptable calibration by the calibration belt (*P* = .71) and the Hosmer-Lemeshow goodness-of-fit test (*P* = .63) in the test cohort (n = 5939) (eFigure 1 in the Supplement). Stratifying the cohort by maximum Pao_2_ values demonstrated that observed mortality surpassed predicted mortality in the 50– to 99–mm Hg (6.7-13.2 kPa) bin, as well as in all bins 300 mm Hg (40 kPa) or more (Figure).

**Figure.**
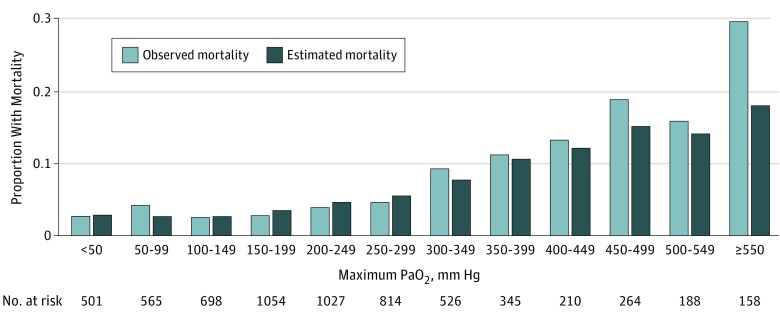
Proportions of Observed and Estimated Mortality for Included Patients Grouped by Maximum Pao_2_ Level During Pediatric Intensive Care Unit Hospitalization Modified Pediatric Logistic Organ Dysfunction-2 score was used as the measure.

Table 2 presents the results of univariable and multivariable logistic regression. After adjusting for significant covariates, hyperoxemia was associated with in-hospital mortality (aOR, 1.78; 95% CI, 1.36-2.33; *P* < .001), as was the m-PELOD-2 score (aOR, 2.63; 95% CI, 2.44-2.84; *P* < .001). The results of a sensitivity analysis assessing possible residual confounding indicated that an unmeasured, binary variable would have to be present in 37% of the encounters with severe hyperoxemia and 0% of the remaining cohort to fail to reject the null hypothesis (aOR of severe hyperoxemia, 1.31; 95% CI, 0.99-1.72) (eTables 1-4 in the Supplement).

**Table 2.** Association of the Maximum Pao_2_ Value During Hospitalization and In-Hospital Mortality Before and After Adjustment With the m-PELOD-2 Score

Variable	Univariable Analysis		Multivariable Analysis	
	OR (95% CI)	*P* Value	aOR (95% CI)^a^	*P* Value
Maximum Pao_2_ value^b^				
No hyperoxemia (<300 mm Hg)	1 [Reference]		1 [Reference]	
Hyperoxemia (≥300 mm Hg)	4.45 (3.70-5.61)	<.001	1.75 (1.33-2.29)	<.001
m-PELOD-2 score^c^	2.71 (2.53-2.91)	<.001	2.60 (2.41-2.81)	<.001
Received ECLS	10.68 (7.46-15.30)	<.001	1.36 (0.84-2.21)	.21
No. of ABG measures during encounter	1.01 (1.01-1.01)	<.001	1.00 (1.00-1.00)	.82
Age, d	1.00 (1.00-1.00)	.36	NA	

Abbreviations: ABG, arterial blood gas; aOR, adjusted odds ratio; ECLS, extracorporeal life support; m-PELOD-2, modified Pediatric Logistic Organ Dysfunction-2; NA, not applicable; OR, odds ratio.

^a^ Included variables of duration of hyperoxemia, m-PELOD-2 score, provision of ECLS, and number of ABG measures per encounter.

^b^ For Pao_2_ level greater than or equal to 300 mm Hg, the kilopascal value is 40 kPa.

^c^ The m-PELOD-2 scores range from 0 to 31, with 0 indicating no organ dysfunction and 31 indicating the greatest amount of organ dysfunction as quantified by the score.

There were 3464 encounters with at least 3 Pao_2_ values separated by at least 3 hours each, of which 2211 encounters (63.8%) did not have documented severe hyperoxemia. There were 816 encounters (23.6%) with 1 severely hyperoxemic Pao_2_ value, 236 encounters (6.8%) with 2, and 201 encounters (5.8%) with 3 or more. In-hospital mortality was 127 of 2211 deaths (5.7%) among encounters without any severely hyperoxemic Pao_2_ values, 102 of 816 deaths (12.5%) among encounters with 1 severely hyperoxemic Pao_2_ value, 53 of 236 deaths (22.5%) among encounters with 2 severely hyperoxemic Pao_2_ values, and 75 of 201 deaths (37.3%) among encounters with 3 or more severely hyperoxemic Pao_2_ values (eTable 5 in the Supplement). In multivariable analysis, the presence of 1 (aOR, 1.47; 95% CI, 1.05-2.08; *P* = .03), 2 (aOR, 2.01; 1.27-3.18; *P* = .002), or 3 or more (aOR, 2.53; 95% CI, 1.62-3.94; *P* < .001) severely hyperoxemic Pao_2_ values in patients were independently associated with in-hospital mortality compared with patients without documented severe hyperoxemia, after adjusting for illness severity, ECLS, and the number of Pao_2_ values obtained during hospitalization (Table 3).

**Table 3.** Association of the Number of Severely Hyperoxemic Pao_2_ Values and In-Hospital Mortality Among Encounters With at Least 3 Pao_2_ Measurements at Least 3 Hours Apart

Variable	Univariable Analysis		Multivariable Analysis	
	OR (95% CI)	*P* Value	aOR (95% CI)^a^	*P* Value
No. of hyperoxemic Pao_2_ values				
No hyperoxemia^b^	1 [Reference]		1 [Reference]	
1 Pao_2_ value ≥300 mm Hg	2.34 (1.78-3.08)	<.001	1.47 (1.05-2.08)	.03
2 Pao_2_ values ≥300 mm Hg	4.75 (3.33-6.77)	<.001	2.01 (1.27-3.18)	.003
≥3 Pao_2_ values ≥300 mm Hg	9.77 (6.97-13.69)	<.001	2.53 (1.62-3.94)	<.001
m-PELOD-2 score^c^	2.66 (2.45-2.89)	<.001	2.53 (2.32-2.76)	<.001
Received ECLS	6.80 (4.69-9.86)	<.001	1.40 (0.84-2.31)	.13
No. of ABG measures during encounter	1.01 (1.01-1.01)	<.001	1.00 (1.00-1.00)	.85
Age, d	1.00 (1.00-1.00)	.20	NA	

Abbreviations: ABG, arterial blood gas; aOR, adjusted odds ratio; ECLS, extracorporeal life support; m-PELOD-2, modified Pediatric Logistic Organ Dysfunction-2; NA, not applicable; OR, odds ratio.

^a^ Included variables of number of hyperoxemic PaO2 values, m-PELOD-2 score, provision of ECLS, and number of ABG measures per encounter.

^b^ For Pao_2_ level greater than or equal to 300 mm Hg, the kilopascal value is 40 kPa.

^c^ The m-PELOD-2 scores range from 0 to 31, with 0 indicating no organ dysfunction and 31 indicating the greatest amount of organ dysfunction as quantified by the score.

Comparing groups of patients with 1, 2, or 3 severely hyperoxemic Pao_2_ values during hospitalization, the median duration between admission time and the first severely hyperoxemic Pao_2_ value was not significantly different. In patients with at least 2 events of severe hyperoxemia, the median durations between the first Pao_2_ and second Pao_2_ values in encounters with 2 (6.2 hours; IQR, 3.8-29.6 hours) or 3 or more (4.8 hours; IQR, 3.8-9.5 hours) severely hyperoxemic Pao_2_ measurements were significantly different (*P* = .04). In patients with 3 severely hyperoxemic Pao_2_ values, the median duration between the first and second severely hyperoxemic Pao_2_ values (median, 4.8 hours; IQR, 3.8-9.5 hours) and second and third severely hyperoxemic gas values (5.1 hours; IQR, 3.8-20.9 hours) were similar (*P* = .30).

### Post Hoc Analyses

Analyses accounting for multiple encounters in the same patient during the inclusion period using only the last available patient encounter (n = 4432) demonstrated results consistent with those of the primary analysis (eTable 6 and eTable 7 in the Supplement), as did the generalized estimating equations logistic regression models clustered by patient identifier (eTable 8 and eTable 9 in the Supplement). Univariable ROC analysis of maximum Pao_2_ value as a predictor of mortality demonstrated an AUROC value of 0.71 and a Youden cut point of 302 mm Hg (40.2 kPa) (eFigure 3 in the Supplement). Using alternative cut point selection methods, the Pao_2_ thresholds ranged from 267 to 641 mm Hg (35.6-85.5 kPa) (eTable 10 in the Supplement). A stepwise increase in the aOR for mortality was observed between severe hyperoxemia and extreme hyperoxemia in a multivariable logistic regression model (eTable 11 in the Supplement). In the MARS model, the association between maximum Pao_2_ value and mortality was pronounced above a threshold of approximately 545 mm Hg (72.7 kPa). Plots demonstrating the splines for each independent variable in the MARS model are presented in eFigure 2 in the Supplement. The AUROC of the MARS model was comparable to the AUROC of the multivariable logistic regression model from Table 2 (0.94 vs 0.93; *P* = .05).

There were 1782 encounters with at least 3 Pao_2_ measurements per day for the initial 3 days of arterial line placement and 232 patients (13.0%) died during hospitalization. The median AUC of PaO_2_ values over 3 days was 8300 mm Hg (IQR, 6828-10 019 mm Hg). The AUC was higher in patients with in-hospital mortality compared with those without in-hospital mortality (9002 [IQR, 7245-11 495]; vs 8215 [IQR, 6792-9861] mm Hg; *P* < .001). Plots derived from the nonparametric MARS model incorporating AUC as a continuous variable with the covariates m-PELOD-2 score, use of ECLS, number of Pao_2_ values, and age demonstrated an association between in-hospital mortality and rising AUC above a threshold of approximately 8355 mm Hg (1113 kPa) over the initial 3 days of arterial line placement (eFigure 4 in the Supplement). A wide range of Pao_2_ values was observed within 20 minutes of documented Spo_2_ and Fio_2_ values; therefore, we did not attempt to use measurements of Spo_2_ and Fio_2_ as surrogates for arterial oxygen tension (eTable 12 in the Supplement).

## Discussion

In this large cohort study examining encounters in a quaternary PICU over 10 years, severe hyperoxemia, defined as a Pao_2_ level of 300 mm Hg (40 kPa) or higher, was independently associated with in-hospital mortality after controlling for a calibrated measure of illness severity, the use of ECLS, and the total number of obtained Pao_2_ measurements. In addition, an exposure-response association was observed, with increasing odds of mortality evident with 1, 2, and 3 or more severely hyperoxemic Pao_2_ values. A sensitivity analysis indicates that these results are robust, as an unmeasured, binary confounder with an effect size of an aOR of 2 would need to be present in more than one-third of encounters with severe hyperoxemia, and not present in any of the nonhyperoxemic encounters, for severe hyperoxemia not to be associated with mortality. Findings from our post hoc analyses are consistent with our initial results.

Our finding of an association between severe hyperoxemia and poor outcome fits into a large body of adult data suggesting a detrimental outcome of high Pao_2_ levels.^17^ In a prospective study of 280 adults, early hyperoxemia was associated with poor neurologic outcomes.^14^ Another study of 6326 adults following cardiac arrest identified an independent association between hyperoxemia and mortality.^12^ Similar studies of pediatric patients have not consistently demonstrated comparable findings, although these have been limited by smaller sample sizes. In one of the largest studies of children after cardiac arrest that included 1875 patients, a first-measured Pao_2_ value of 300 mm Hg (40 kPa) or higher following ICU admission was independently associated with mortality.^26^ In addition, 2 observational studies report associations between admission hyperoxemia and mortality among diagnostically diverse, critically ill children.^23,24^ Raman et al^24^ identified a U-shaped association between admission Pao_2_ values and mortality among 7410 children admitted to a PICU, after adjusting for age, sex, and an m-PELOD 2 score that excluded Pao_2_/Fio_2_ ratio measurements to control for illness severity. Risk of mortality was observed to rise below a Pao_2_ level of 188 mm Hg (25.1 kPa) and above 300 mm Hg (40 kPa). Numa et al^23^ observed among 1447 PICU patients that an admission Pao_2_ value greater than 250 mm Hg (33.3 kPa) was associated with 2.66 increased odds of death after adjusting for illness severity using the Pediatric Index of Mortality-3.

The association between Pao_2_ level and in-hospital mortality identified in our own and other observational studies may reflect confounding by indication. Higher concentrations of supplemental oxygen are commonly administered during resuscitation, with the sickest patients often receiving an Fio_2_ of 1.0, via either a nonrebreather facemask or positive pressure ventilation. Severity of illness measurements, such as versions of the Pediatric Index of Mortality or the Pediatric Risk of Mortality score, are conventionally used to control for acuity of the patient’s condition when examining whether a factor such as hyperoxemia is independently associated with an outcome such as mortality. However, these common severity-of-illness measurements have been developed using multicenter data and typically require recalibration to uniformly control for illness severity when applied to single institution data.^30,31^ In addition, the Pediatric Index of Mortality and Pediatric Risk of Mortality score include only data surrounding PICU admission and therefore do not adequately control for increasing illness severity later during a hospitalization. We calibrated a modified version of the PELOD-2 to our institutional data to generate a predicted mortality risk representative of population-level outcomes at our institution across the full spectrum of illness severity. The m-PELOD-2 incorporated data from the patient’s entire hospitalization, in contrast to admission illness severity measurements, which only incorporate data from a narrower time window surrounding admission.^32,33^

The observation of a possible biological gradient or dose-response with increasing severe hyperoxemic events in the present study is consistent with the biological mechanisms postulated to underlie the harm conferred by hyperoxemia. For patients with multiple hyperoxemic Pao_2_ values, these measures were most commonly separated by several hours. Periods of prolonged hyperoxemia may have an aggravated proinflammatory response^34^ and lead to depletion of endogenous free-radical scavenger systems.^35^ The study by van Zellem et al^21^ of children receiving mild therapeutic hypothermia following cardiac arrest suggested a possible benefit of cumulative oxygen exposure during the first 24 hours of admission, in which oxygen exposure was measured by an estimated AUC based on Pao_2_ values using the trapezoid method. In that study, the median AUC for the first 24 hours was 3264.8 mm Hg (136 mm Hg per hour) in survivors vs 3119.9 mm Hg (130 mm Hg per hour) in nonsurvivors, which was not a statistically significant difference. A high AUC could be observed in patients without any Pao_2_ values above 200 mm Hg (26.7 kPa), and results were only nominally significant in multivariable analysis without accounting for multiple testing.

Although true causal inference will require rigorous, prospective evaluation of hyperoxemia as it relates to mortality, the existing evidence, coupled with the biological plausibility of the deleterious effects of hyperoxemia, continues to warrant consideration in the design of guidelines for both resuscitation and ongoing support of critically ill children. The 2015 update of the American Heart Association guidelines for pediatric advanced life support recommend starting resuscitation with an Fio_2_ level of 1.0, then weaning when able to target an oxyhemoglobin saturation of 94% to 99%.^36^ Similarly, the European Resuscitation Council recommends maintaining oxyhemoglobin saturations in the range of 94% to 98%.^37^ Recognition of possible harm caused by hyperoxemia in newborns^38,39,40^ prompted changes to resuscitation guidelines,^41,42^ although a prospective, randomized clinical trial of lower vs higher target oxygen saturations in neonates demonstrated an increased risk of mortality in the target oxygenation range of 85% to 89% compared with 91% to 95%.^43^

Our sensitivity analyses indicate that a beneficial association with hyperoxemia could have been masked by an unmeasured confounder with an aOR of 5 that was present in approximately 40% of encounters with hyperoxemia and 0% of the encounters with normoxemia. Prospective data are needed to determine whether targeting normoxemia and avoiding both hypoxemia and hyperoxemia is the safest approach to provide ongoing life support for critically ill children without cyanotic heart disease, or exceptional disease states, such as cyanide poisoning or high concentrations of carboxyhemoglobinemia, One pilot, multicenter, randomized trial examined conservative vs liberal oxygenation management among critically ill children and demonstrated the feasibility of a prospective study using Spo_2_ and Fio_2_ as study targets.^44^ In contrast, Spo_2_ was not a reliable surrogate of Pao_2_ in our study.

### Limitations

This study is limited by its retrospective observational design. Because we only included maximum Pao_2_ values, we did not assess for an association between hypoxemia and mortality, and a lower acceptable threshold of Pao_2_ cannot be identified in the present analysis. The timing and frequency of Pao_2_ values were determined by the treating clinical team, with sicker patients and patients with longer hospitalizations likely contributing to sampling bias. We attempted to control for this possible bias by including the total number of Pao_2_ values obtained per encounter in our regression models. Furthermore, it is unclear in the present study whether the median times of more than 4 hours between the first, second, and third hyperoxemic Pao_2_ value measurements signify periods of protracted exposure to hyperoxia or shorter discrete events. Sensitivity analyses of unmeasured confounding modeled the theoretic effect of a single dichotomous variable; however, in the dynamic context of disease and patient care, confounding may occur as an aggregate of transient exposures. We did not distinguish different categories of disease in the present cohort and therefore may have missed a beneficial effect of hyperoxemia in certain disease states, such as traumatic brain injury.^45^ Although our sensitivity analysis appears to indicate that the present results are robust, residual confounding remains possible and would be optimally addressed with a prospective, randomized trial design.

## Conclusions

Data derived from a large cohort of patients admitted to a PICU suggest that severe hyperoxemia was independently associated with in-hospital mortality. In addition, a greater number of measured severe hyperoxemic Pao_2_ values during hospitalization appear to be associated with increased odds of in-hospital mortality. These findings, while in need of prospective validation, seem to support an association between severe hyperoxemia and poor outcomes among critically ill children and adolescents. Future guidelines for the ongoing support of critically ill children may account for the possible deleterious effects of supratherapeutic oxygen levels in this population.

## Notes

Supplement. **eFigure 1.** Receiver Operating Characteristic Curves and Calibration Belts for m-PELOD 2 Initially and Following Re-Calibration to Data Set **eFigure 2.** Multivariate Adaptive Regression Splines Using the Maximum Pao2 for Each Encounter With Relevant Covariates. for Each Plot **eFigure 3.** Threshold Analysis for Maximum Pao_2_ During Encounter Using Unadjusted ROC Curve **eFigure 4.** Multivariate Adaptive Regression Splines Using the Pao2 AUC for Encounters With at Least 3 Pao2 Values per Day, With Relevant Covariates **eTable 1.** Sensitivity Analysis Demonstrating the Effect of a Hypothetical Unmeasured Risk Factor (Confounder) on the Adjusted Odds Ratio of the Primary Exposure of Interest (Hyperoxemia) on In-Patient Mortality at an Adjusted Odds Ratio of the Confounder of 2 and at Varying Prevalence in the Exposed and Unexposed Groups **eTable 2.** Sensitivity Analysis Demonstrating the Effect of a Hypothetical Unmeasured Risk Factor (Confounder) on the Adjusted Odds Ratio of the Primary Exposure of Interest (Hyperoxemia) on In-Patient Mortality at an Adjusted Odds Ratio of the Confounder of 3 and at Varying Prevalence in the Exposed and Unexposed Groups **eTable 3.** Sensitivity Analysis Demonstrating the Effect of a Hypothetical Unmeasured Risk Factor (Confounder) on the Adjusted Odds Ratio of the Primary Exposure of Interest (Hyperoxemia) on In-Patient Mortality at an Adjusted Odds Ratio of the Confounder of 4 and at Varying Prevalence in the Exposed and Unexposed Groups **eTable 4.** Sensitivity Analysis Demonstrating the Effect of a Hypothetical Unmeasured Risk Factor (Confounder) on the Adjusted Odds Ratio of the Primary Exposure of Interest (Hyperoxemia) on In-Patient Mortality at an Adjusted Odds Ratio of the Confounder of 5 and at Varying Prevalence in the Exposed and Unexposed Groups **eTable 5.** Descriptive Data for Encounters Categorized by Number of Pao_2_ Values Corresponding With Hyperoxia Among Patients With ≥3 Pao_2_ Measurements Collected ≥3 Hours Apart **eTable 6.** Association of the Maximum Pao_2_ During Hospitalization and In-Hospital Mortality Before and After Adjustment With the M-PELOD 2 Score, Retaining Only the Last Encounter for Each Patient During Inclusion Period (N = 4432 Subjects) **eTable 7.** Association of the Number of Pao_2_ Measurements With Hyperoxemia and In-Hospital Mortality, Retaining Only the Last Encounter for Each Patient During Inclusion Period (N = 4432) Subjects **eTable 8.** Association of the Maximum Pao_2_ During Hospitalization and In-Hospital Mortality Using Generalized Estimating Equations Clustered by Patient Identifier **eTable 9.** Association of the Number of Pao_2_ Measurements With Hyperoxemia and In-Hospital Mortality Among Those Patients With at Least Three Pao_2_ Measurements at Least Three Hours Apart as a Sensitivity Analysis Using Generalized Estimating Equations to Account for Clustering Around Subjects **eTable 10.** Optimal Thresholds Derived From Receiver Operator Curve Analysis **eTable 11.** Multivariable Logistic Regression Model Examining Additional Thresholds of Maximum Pao_2_ Values **eTable 12.** Proportions of Pao_2_ Corresponding With Hyperoxia at Different Fractions of Inspired Oxygen (Fio_2_) When Oxygen Saturation (Spo_2_) Was 100% From All Encounters in Which an ABG Was Done Within 20 Minutes of A Charted Fio_2_ and Spo_2_

## References

[1] O’Driscoll BR, Howard LS, Earis J, Mak V; British Thoracic Society Emergency Oxygen Guideline Group; BTS Emergency Oxygen Guideline Development Group BTS guideline for oxygen use in adults in healthcare and emergency settings. Thorax. 2017;72(suppl 1):-. doi:10.1136/thoraxjnl-2016-209729 28883921 PMC5531304

[2] Hale KE, Gavin C, O’Driscoll BR Audit of oxygen use in emergency ambulances and in a hospital emergency department. Emerg Med J. 2008;25(11):773-776. doi:10.1136/emj.2008.059287 18955625

[3] Smith JL The pathological effects due to increase of oxygen tension in the air breathed. J Physiol. 1899;24(1):19-35. doi:10.1113/jphysiol.1899.sp000746 16992479 PMC1516623

[4] Nagato AC, Bezerra FS, Lanzetti M, Time course of inflammation, oxidative stress and tissue damage induced by hyperoxia in mouse lungs. Int J Exp Pathol. 2012;93(4):269-278. doi:10.1111/j.1365-2613.2012.00823.x 22804763 PMC3444983

[5] Fracica PJ, Knapp MJ, Piantadosi CA, Responses of baboons to prolonged hyperoxia: physiology and qualitative pathology. J Appl Physiol (1985). 1991;71(6):2352-2362. doi:10.1152/jappl.1991.71.6.2352 1778933

[6] Farquhar H, Weatherall M, Wijesinghe M, Systematic review of studies of the effect of hyperoxia on coronary blood flow. Am Heart J. 2009;158(3):371-377. doi:10.1016/j.ahj.2009.05.037 19699859

[7] Lodato RF Decreased O2 consumption and cardiac output during normobaric hyperoxia in conscious dogs. J Appl Physiol (1985). 1989;67(4):1551-1559. doi:10.1152/jappl.1989.67.4.1551 2793757

[8] Mantell LL, Lee PJ Signal transduction pathways in hyperoxia-induced lung cell death. Mol Genet Metab. 2000;71(1-2):359-370. doi:10.1006/mgme.2000.3046 11001828

[9] Barazzone C, Horowitz S, Donati YR, Rodriguez I, Piguet PF Oxygen toxicity in mouse lung: pathways to cell death. Am J Respir Cell Mol Biol. 1998;19(4):573-581. doi:10.1165/ajrcmb.19.4.3173 9761753

[10] Waxman AB, Einarsson O, Seres T, Targeted lung expression of interleukin-11 enhances murine tolerance of 100% oxygen and diminishes hyperoxia-induced DNA fragmentation. Chest. 1999;116(1)(suppl):8S-9S. doi:10.1378/chest.116.suppl_1.8S 10424559

[11] Elmer J, Scutella M, Pullalarevu R, ; Pittsburgh Post-Cardiac Arrest Service (PCAS) The association between hyperoxia and patient outcomes after cardiac arrest: analysis of a high-resolution database. Intensive Care Med. 2015;41(1):49-57. doi:10.1007/s00134-014-3555-6 25472570 PMC4337386

[12] Kilgannon JH, Jones AE, Shapiro NI, ; Emergency Medicine Shock Research Network (EMShockNet) Investigators Association between arterial hyperoxia following resuscitation from cardiac arrest and in-hospital mortality. JAMA. 2010;303(21):2165-2171. doi:10.1001/jama.2010.707 20516417

[13] Janz DR, Hollenbeck RD, Pollock JS, McPherson JA, Rice TW Hyperoxia is associated with increased mortality in patients treated with mild therapeutic hypothermia after sudden cardiac arrest. Crit Care Med. 2012;40(12):3135-3139. doi:10.1097/CCM.0b013e3182656976 22971589 PMC3502652

[14] Roberts BW, Kilgannon JH, Hunter BR, Association between early hyperoxia exposure after resuscitation from cardiac arrest and neurological disability: prospective multicenter protocol-directed cohort study. Circulation. 2018;137(20):2114-2124. doi:10.1161/CIRCULATIONAHA.117.032054 29437118 PMC6370332

[15] Helmerhorst HJF, Roos-Blom M-J, van Westerloo DJ, Abu-Hanna A, de Keizer NF, de Jonge E Associations of arterial carbon dioxide and arterial oxygen concentrations with hospital mortality after resuscitation from cardiac arrest. Crit Care. 2015;19:348. doi:10.1186/s13054-015-1067-6 26415731 PMC4587673

[16] Vaahersalo J, Bendel S, Reinikainen M, ; FINNRESUSCI Study Group Arterial blood gas tensions after resuscitation from out-of-hospital cardiac arrest: associations with long-term neurologic outcome. Crit Care Med. 2014;42(6):1463-1470. doi:10.1097/CCM.0000000000000228 24557423

[17] Chu DK, Kim LH-Y, Young PJ, Mortality and morbidity in acutely ill adults treated with liberal versus conservative oxygen therapy (IOTA): a systematic review and meta-analysis. Lancet. 2018;391(10131):1693-1705. doi:10.1016/S0140-6736(18)30479-3 29726345

[18] Guerra-Wallace MM, Casey FL III, Bell MJ, Fink EL, Hickey RW Hyperoxia and hypoxia in children resuscitated from cardiac arrest. Pediatr Crit Care Med. 2013;14(3):e143-e148. doi:10.1097/PCC.0b013e3182720440 23392367 PMC3654405

[19] López-Herce J, Del Castillo J, Matamoros M, ; Iberoamerican Pediatric Cardiac Arrest Study Network RIBEPCI Factors associated with mortality in pediatric in-hospital cardiac arrest: a prospective multicenter multinational observational study. Intensive Care Med. 2013;39(2):309-318. doi:10.1007/s00134-012-2709-7 23184036

[20] Bennett KS, Clark AE, Meert KL, ; Pediatric Emergency Care Medicine Applied Research Network Early oxygenation and ventilation measurements after pediatric cardiac arrest: lack of association with outcome. Crit Care Med. 2013;41(6):1534-1542. doi:10.1097/CCM.0b013e318287f54c 23552509 PMC3683244

[21] van Zellem L, de Jonge R, van Rosmalen J, Reiss I, Tibboel D, Buysse C High cumulative oxygen levels are associated with improved survival of children treated with mild therapeutic hypothermia after cardiac arrest. Resuscitation. 2015;90:150-157. doi:10.1016/j.resuscitation.2014.12.013 25576438

[22] Del Castillo J, López-Herce J, Matamoros M, ; Iberoamerican Pediatric Cardiac Arrest Study Network RIBEPCI Hyperoxia, hypocapnia and hypercapnia as outcome factors after cardiac arrest in children. Resuscitation. 2012;83(12):1456-1461. doi:10.1016/j.resuscitation.2012.07.019 22841610

[23] Numa A, Aneja H, Awad J, Admission hyperoxia is a risk factor for mortality in pediatric intensive care. Pediatr Crit Care Med. 2018;19(8):699-704. doi:10.1097/PCC.0000000000001630 29927878

[24] Raman S, Prince NJ, Hoskote A, Ray S, Peters MJ Admission Pao_2_ and mortality in critically ill children: a cohort study and systematic review. Pediatr Crit Care Med. 2016;17(10):e444-e450. doi:10.1097/PCC.0000000000000905 27509363

[25] von Elm E, Altman DG, Egger M, Pocock SJ, Gøtzsche PC, Vandenbroucke JP; STROBE Initiative The Strengthening the Reporting of Observational Studies in Epidemiology (STROBE) statement: guidelines for reporting observational studies. J Clin Epidemiol. 2008;61(4):344-349. doi:10.1016/j.jclinepi.2007.11.008 18313558

[26] Ferguson LP, Durward A, Tibby SM Relationship between arterial partial oxygen pressure after resuscitation from cardiac arrest and mortality in children. Circulation. 2012;126(3):335-342. doi:10.1161/CIRCULATIONAHA.111.085100 22723307

[27] Leteurtre S, Duhamel A, Salleron J, Grandbastien B, Lacroix J, Leclerc F; Groupe Francophone de Réanimation et d’Urgences Pédiatriques (GFRUP) PELOD-2: an update of the PEdiatric logistic organ dysfunction score. Crit Care Med. 2013;41(7):1761-1773. doi:10.1097/CCM.0b013e31828a2bbd 23685639

[28] Horvat C, Ogoe H, Kantawala S, Development and performance of electronic Pediatric Risk of Mortality and Pediatric Logistic Organ Dysfunction Automated Acuity scores [published online August 8, 2019]. Pediatr Crit Care Med. doi:10.1097/PCC.0000000000001998. Accessed August 8, 2019. PMC7115250 31397827

[29] Friedman JH Multivariate adaptive regression splines. Ann Stat. 1991;19(1):1-67. doi:10.1214/aos/1176347963 8548103

[30] Pollack MM, Holubkov R, Funai T, ; Eunice Kennedy Shriver National Institute of Child Health and Human Development Collaborative Pediatric Critical Care Research Network Simultaneous prediction of new morbidity, mortality, and survival without new morbidity from pediatric intensive care: a new paradigm for outcomes assessment. Crit Care Med. 2015;43(8):1699-1709. doi:10.1097/CCM.0000000000001081 25985385 PMC4657566

[31] Marcin JP, Pollack MM Review of the acuity scoring systems for the pediatric intensive care unit and their use in quality improvement. J Intensive Care Med. 2007;22(3):131-140. doi:10.1177/0885066607299492 17562737

[32] Straney L, Clements A, Parslow RC, ; ANZICS Paediatric Study Group and the Paediatric Intensive Care Audit Network Paediatric Index of Mortality 3: an updated model for predicting mortality in pediatric intensive care. Pediatr Crit Care Med. 2013;14(7):673-681. doi:10.1097/PCC.0b013e31829760cf 23863821

[33] Pollack MM, Holubkov R, Funai T, ; Eunice Kennedy Shriver National Institute of Child Health and Human Development Collaborative Pediatric Critical Care Research Network The Pediatric Risk of Mortality score: update 2015. Pediatr Crit Care Med. 2016;17(1):2-9. doi:10.1097/PCC.0000000000000558 26492059 PMC5048467

[34] Helmerhorst HJF, Schouten LRA, Wagenaar GTM, Hyperoxia provokes a time- and dose-dependent inflammatory response in mechanically ventilated mice, irrespective of tidal volumes. Intensive Care Med Exp. 2017;5(1):27. doi:10.1186/s40635-017-0142-5 28550659 PMC5446430

[35] Turrens JF Mitochondrial formation of reactive oxygen species. J Physiol. 2003;552(pt 2):335-344. doi:10.1113/jphysiol.2003.049478 14561818 PMC2343396

[36] de Caen AR, Berg MD, Chameides L, Part 12: Pediatric advanced life support: 2015 American Heart Association Guidelines update for cardiopulmonary resuscitation and emergency cardiovascular care. Circulation. 2015;132(18)(suppl 2):S526-S542. doi:10.1161/CIR.0000000000000266 26473000 PMC6191296

[37] Maconochie IK, Bingham R, Eich C, ; Paediatric Life Support Section Collaborators European Resuscitation Council Guidelines for Resuscitation 2015: section 6. paediatric life support. Resuscitation. 2015;95:223-248. doi:10.1016/j.resuscitation.2015.07.028 26477414

[38] Vento M, Moro M, Escrig R, Preterm resuscitation with low oxygen causes less oxidative stress, inflammation, and chronic lung disease. Pediatrics. 2009;124(3):e439-e449. doi:10.1542/peds.2009-0434 19661049

[39] Davis PG, Tan A, O’Donnell CPF, Schulze A Resuscitation of newborn infants with 100% oxygen or air: a systematic review and meta-analysis. Lancet. 2004;364(9442):1329-1333. doi:10.1016/S0140-6736(04)17189-4 15474135

[40] Tan A, Schulze A, O’Donnell CPF, Davis PG Air versus oxygen for resuscitation of infants at birth. Cochrane Database Syst Rev. 2005;(2):CD002273. doi:10.1002/14651858.CD002273.pub3 15846632 PMC7017642

[41] Wyckoff MH, Aziz K, Escobedo MB, Part 13: Neonatal Resuscitation: 2015 American Heart Association Guidelines Update for Cardiopulmonary Resuscitation and Emergency Cardiovascular Care. Circulation. 2015;132(18)(suppl 2):S543-S560. doi:10.1161/CIR.0000000000000267 26473001

[42] Sweet DG, Carnielli V, Greisen G, European consensus guidelines on the management of respiratory distress syndrome—2016 update. Neonatology. 2017;111(2):107-125. doi:10.1159/000448985 27649091

[43] Carlo WA, Finer NN, Walsh MC, et al; SUPPORT Study Group of the Eunice Kennedy Shriver NICHD Neonatal Research Network. Target ranges of oxygen saturation in extremely preterm infants N Engl J Med. 2010;362(21):1959-1969. doi:10.1056/NEJMoa0911781 20472937 PMC2891970

[44] Peters MJ, Jones GAL, Wiley D, ; Oxy-PICU Investigators for the Paediatric Intensive Care Society Study Group (PICS-SG) Conservative versus liberal oxygenation targets in critically ill children: the randomised multiple-centre pilot Oxy-PICU trial. Intensive Care Med. 2018;44(8):1240-1248. doi:10.1007/s00134-018-5232-7 29868973

[45] Asher SR, Curry P, Sharma D, Survival advantage and Pao_2_ threshold in severe traumatic brain injury. J Neurosurg Anesthesiol. 2013;25(2):168-173. doi:10.1097/ANA.0b013e318283d350 23343758

