# Supplementary material for: Association of Severe Hyperoxemia Events and Mortality Among Patients Admitted to a Pediatric Intensive Care Unit
Source: JAMA Netw Open. 2019 Aug 21;2(8):e199812. doi: 10.1001/jamanetworkopen.2019.9812 (PMC6707098; doi:10.1001/jamanetworkopen.2019.9812)
Supplement: Supplement. — eFigure 1. Receiver Operating Characteristic Curves and Calibration Belts for m-PELOD 2 Initially and Following Re-Calibration to Data Set eFigure 2. Multivariate Adaptive Regression Splines Using the Maximum Pao2 for Each Encounter With Relevant Covariates. for Each Plot eFigure 3. Threshold Analysis for Maximum Pao2 During Encounter Using Unadjusted ROC Curve eFigure 4. Multivariate Adaptive Regression Splines Using the Pao2 AUC for Encounters With at Least 3 Pao2 Values per Day, With Relevant Covariates eTable 1. Sensitivity Analysis Demonstrating the Effect of a Hypothetical Unmeasured Risk Factor (Confounder) on the Adjusted Odds Ratio of the Primary Exposure of Interest (Hyperoxemia) on In-Patient Mortality at an Adjusted Odds Ratio of the Confounder of 2 and at Varying Prevalence in the Exposed and Unexposed Groups eTable 2. Sensitivity Analysis Demonstrating the Effect of a Hypothetical Unmeasured Risk Factor (Confounder) on the Adjusted Odds Ratio of the Primary Exposure of Interest (Hyperoxemia) on In-Patient Mortality at an Adjusted Odds Ratio of the Confounder of 3 and at Varying Prevalence in the Exposed and Unexposed Groups eTable 3. Sensitivity Analysis Demonstrating the Effect of a Hypothetical Unmeasured Risk Factor (Confounder) on the Adjusted Odds Ratio of the Primary Exposure of Interest (Hyperoxemia) on In-Patient Mortality at an Adjusted Odds Ratio of the Confounder of 4 and at Varying Prevalence in the Exposed and Unexposed Groups eTable 4. Sensitivity Analysis Demonstrating the Effect of a Hypothetical Unmeasured Risk Factor (Confounder) on the Adjusted Odds Ratio of the Primary Exposure of Interest (Hyperoxemia) on In-Patient Mortality at an Adjusted Odds Ratio of the Confounder of 5 and at Varying Prevalence in the Exposed and Unexposed Groups eTable 5. Descriptive Data for Encounters Categorized by Number of Pao2 Values Corresponding With Hyperoxia Among Patients With ≥3 Pao2 Measurements Collected ≥3 Hours Apart eTable 6. Association of t [file jamanetwopen-2-e199812-s001.pdf]

Supplementary Online Content

Ramgopal S, Dezfulian C, Hickey RW, et al. Association of severe hyperoxemia events and mortality among patients admitted to a pediatric intensive care unit. *JAMA Netw Open*. 2019;2(8):e199812. doi:10.1001/jamanetworkopen.2019.9812

eFigure 1. Receiver Operating Characteristic Curves and Calibration Belts for m-PELOD 2 Initially and Following Re-Calibration to Data Set

eFigure 2. Multivariate Adaptive Regression Splines Using the Maximum PaO2 for Each Encounter With Relevant Covariates. for Each Plot

eFigure 3. Threshold Analysis for Maximum PaO2 During Encounter Using Unadjusted ROC Curve

eFigure 4. Multivariate Adaptive Regression Splines Using the PaO2 AUC for Encounters With at Least 3 PaO2 Values per Day, With Relevant Covariates

eTable 1. Sensitivity Analysis Demonstrating the Effect of a Hypothetical Unmeasured Risk Factor (Confounder) on the Adjusted Odds Ratio of the Primary Exposure of Interest (Hyperoxemia) on In-Patient Mortality at an Adjusted Odds Ratio of the Confounder of 2 and at Varying Prevalence in the Exposed and Unexposed Groups

eTable 2. Sensitivity Analysis Demonstrating the Effect of a Hypothetical Unmeasured Risk Factor (Confounder) on the Adjusted Odds Ratio of the Primary Exposure of Interest (Hyperoxemia) on In-Patient Mortality at an Adjusted Odds Ratio of the Confounder of 3 and at Varying Prevalence in the Exposed and Unexposed Groups

eTable 3. Sensitivity Analysis Demonstrating the Effect of a Hypothetical Unmeasured Risk Factor (Confounder) on the Adjusted Odds Ratio of the Primary Exposure of Interest (Hyperoxemia) on In-Patient Mortality at an Adjusted Odds Ratio of the Confounder of 4 and at Varying Prevalence in the Exposed and Unexposed Groups

eTable 4. Sensitivity Analysis Demonstrating the Effect of a Hypothetical Unmeasured Risk Factor (Confounder) on the Adjusted Odds Ratio of the Primary Exposure of Interest (Hyperoxemia) on In-Patient Mortality at an Adjusted Odds Ratio of the Confounder of 5 and at Varying Prevalence in the Exposed and Unexposed Groups

eTable 5. Descriptive Data for Encounters Categorized by Number of Pao_2 Values Corresponding With Hyperoxia Among Patients With ≥3 Pao_2 Measurements Collected ≥3 Hours Apart

eTable 6. Association of the Maximum Pao_2 During Hospitalization and In-Hospital Mortality Before and After Adjustment With the M-PELOD 2 Score, Retaining Only the Last Encounter for Each Patient During Inclusion Period (N=4,432 Subjects)

eTable 7. Association of the Number of Pao_2 Measurements With Hyperoxemia and In-Hospital Mortality, Retaining Only the Last Encounter for Each Patient During Inclusion Period (N=4,432) Subjects

eTable 8. Association of the Maximum Pao_2 During Hospitalization and In-Hospital Mortality Using Generalized Estimating Equations Clustered by Patient Identifier

eTable 9. Association of the Number of Pao_2 Measurements With Hyperoxemia and In-Hospital Mortality Among Those Patients With at Least Three Pao_2 Measurements at Least Three Hours Apart as a Sensitivity Analysis Using Generalized Estimating Equations to Account for Clustering Around Subjects

eTable 10. Optimal Thresholds Derived From Receiver Operator Curve Analysis

eTable 11. Multivariable Logistic Regression Model Examining Additional Thresholds of Maximum Pao_2 Values

eTable 12. Proportions of Pao_2 Corresponding With Hyperoxia at Different Fractions of Inspired Oxygen (Fio_2) When Oxygen Saturation (Spo_2) Was 100% From All Encounters in Which an ABG Was Done Within 20 Minutes of A Charted Fio_2 and Spo_2

This supplementary material has been provided by the authors to give readers additional information about their work.

© 2019 Ramgopal S et al. *JAMA Network Open.*

eFigure 1. Receiver Operating Characteristic Curves and Calibration Belts for m-PELOD 2 (A) Initially and (B) Following Re-Calibration to Data Set

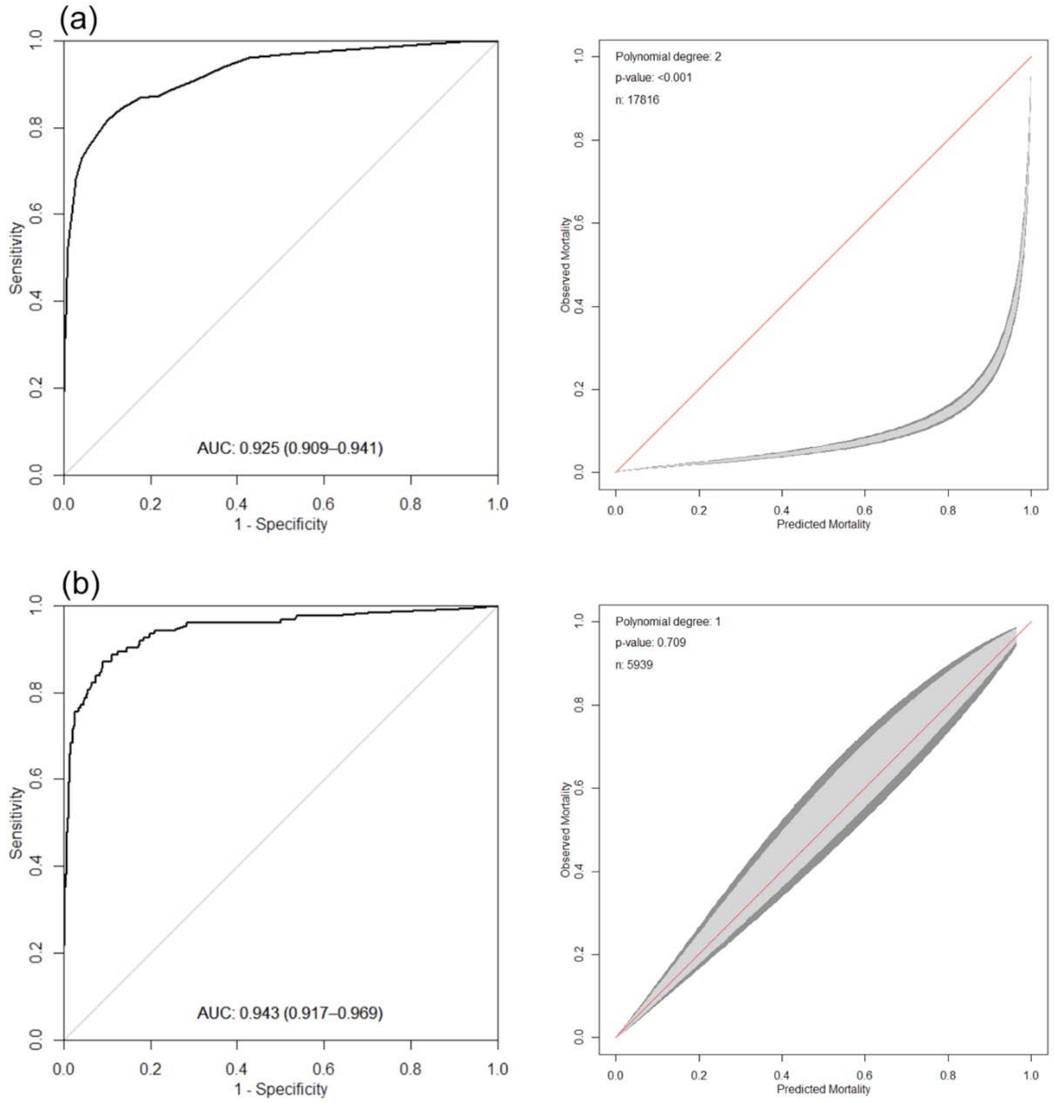

© 2019 Ramgopal S et al. *JAMA Network Open.*

eFigure 2. Multivariate Adaptive Regression Splines Using the Maximum PaO_2 for Each Encounter With Relevant Covariates. for Each Plot .the Y-axis is the probability of outcome (in-hospital mortality).

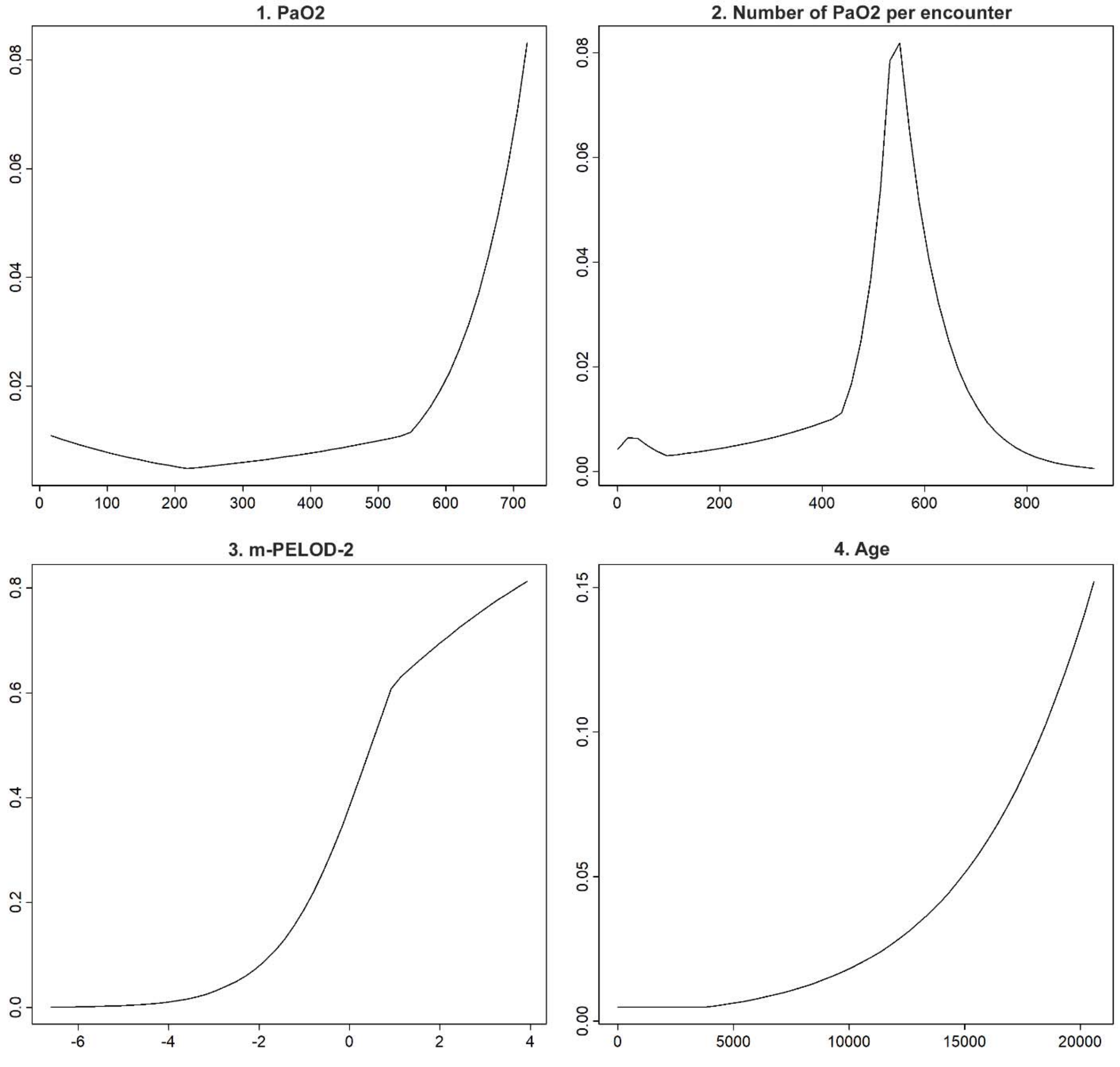

© 2019 Ramgopal S et al. *JAMA Network Open.*

eFigure 3. Threshold analysis for maximum PaO_2 during encounter using unadjusted ROC curve

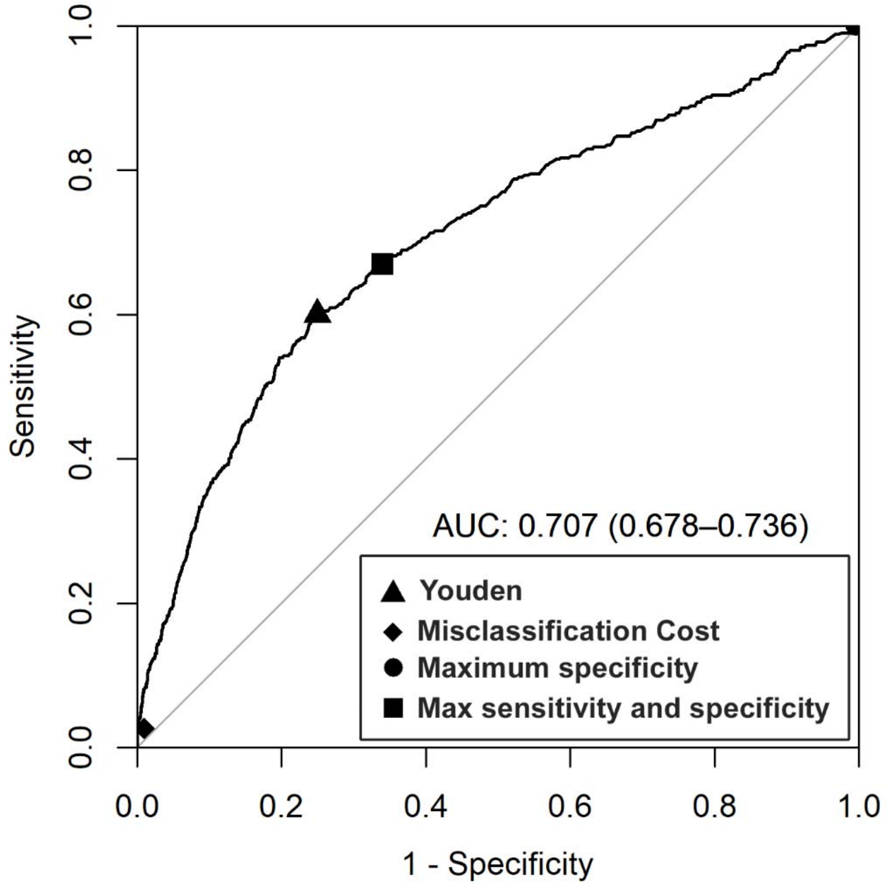

© 2019 Ramgopal S et al. *JAMA Network Open.*

eFigure 4. Multivariate adaptive regression splines using the PaO_2 AUC for encounters with at least 3 PaO_2 values per day, with relevant covariates. for each plot, the y-axis is the probability of outcome (in-hospital mortality).

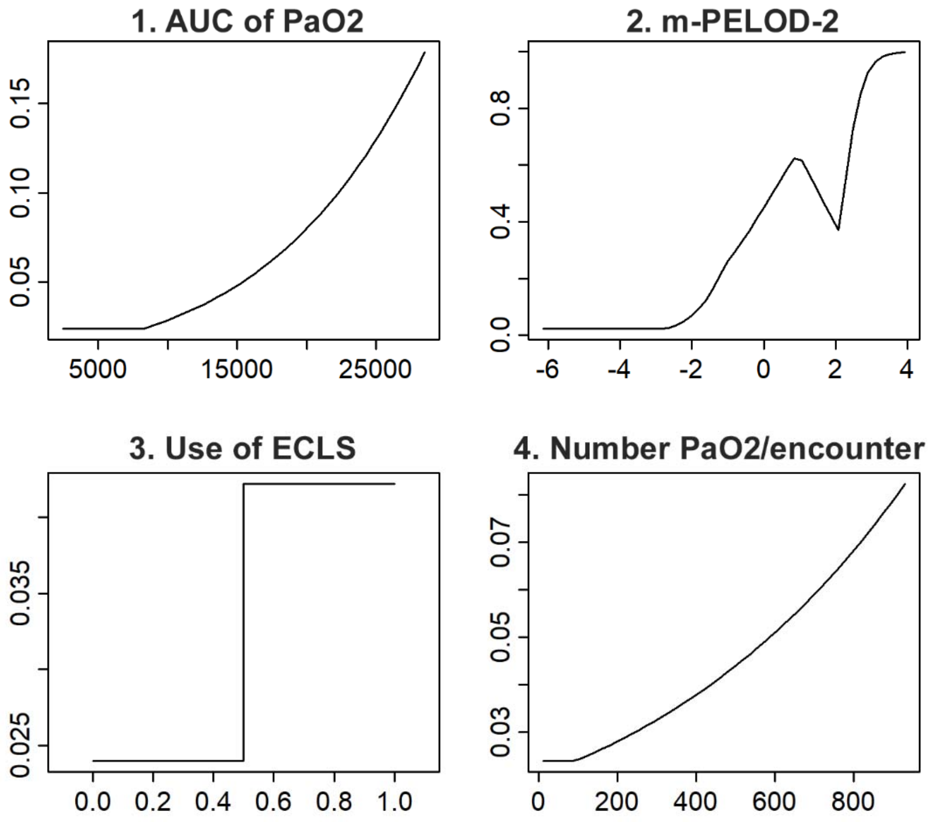

© 2019 Ramgopal S et al. *JAMA Network Open.*

eTable 1. Sensitivity analysis demonstrating the effect of a hypothetical unmeasured risk factor (confounder) on the adjusted odds ratio of the primary exposure of interest (hyperoxemia) on in-patient mortality at an adjusted odds ratio of the confounder of 2 and at varying prevalence in the exposed and unexposed groups. Values in cells represent the adjusted odds ratio of hyperoxia on in-patient mortality with 95% confidence intervals in parenthesis. Cells with bold text are those in which the 95% confidence interval for hyperoxemia crosses or is entirely below one.

	Prevalence in normoxemic group						
Prevalence in hyperoxemic group		**0.0**	**0.2**	**0.4**	**0.6**	**0.8**	**1.0**
	**0.0**	1.766 (1.344, 2.319)	2.119 (1.612, 2.783)	2.472 (1.881-3.246)	2.825 (2.150, 3.710)	3.178 (2.418, 4.174)	3.531 (2.687, 4.638)
	**0.2**	1.471 (1.120, 1.932)	1.766 (1.344, 2.319)	2.060 (1.568, 2.705)	2.354 (1.791, 3.092)	2.694 (2.015, 3.478)	2.943 (2.239, 3.865)
	**0.4**	**1.261 (0.960, 1.656)**	1.513 (1.152, 1.988)	1.766 (1.344, 2.319)	2.018 (1.536, 2.650)	2.270 (1.727, 2.981)	2.522 (1.919, 3.313)
	**0.6**	**1.104 (0.840, 1.449)**	1.324 (1.008, 1.739)	1.545 (1.176, 2.029)	1.766 (1.344, 2.319)	1.986 (1.512, 2.609)	2.207 (1.680, 2.899)
	**0.8**	**0.981 (0.746, 1.288)**	**1.177 (0.896, 1.546)**	1.373 (1.045, 1.804)	1.569 (1.194, 2.061)	1.766 (1.344, 2.319)	1.962 (1.493, 2.576)
	**1.0**	**0.883 (0.672, 1.159)**	**1.059 (0.806, 1.391)**	**1.236 (0.941, 1.623)**	1.413 (1.075, 1.855)	1.589 (1.209, 2.087)	1.766 (1.344, 2.319)

© 2019 Ramgopal S et al. *JAMA Network Open.*

eTable 2. Sensitivity analysis demonstrating the effect of a hypothetical unmeasured risk factor (confounder) on the adjusted odds ratio of the primary exposure of interest (hyperoxemia) on in-patient mortality at an adjusted odds ratio of the confounder of 3 and at varying prevalence in the exposed and unexposed groups. Values in cells represent the adjusted odds ratio of hyperoxia on in-patient mortality with 95% confidence intervals in parenthesis. Cells with bold text are those in which the 95% confidence interval for hyperoxemia crosses or is entirely below one.

Odds ratio=3							
	Prevalence in normoxemic group						
Prevalence in hyperoxemic group		**0.0**	**0.2**	**0.4**	**0.6**	**0.8**	**1.0**
	**0.0**	1.766 (1.344, 2.319)	2.472 (1.881, 3.246)	3.178 (2.418, 4.174)	3.884 (2.956, 5.101)	4.591 (3.493, 6.029)	5.297 (4.031, 6.957)
	**0.2**	**1.261 (0.960, 1.656)**	1.766 (1.344, 2.319)	2.270 (1.727, 2.891)	2.775 (2.111, 3.644)	3.279 (2.495, 4.306)	3.784 (2.879, 4.969)
	**0.4**	**0.981 (0.746, 1.288)**	1.373 (1.045, 1.804)	1.766 (1.344, 2.319)	2.158 (1.642, 2.834)	2.550 (1.941, 3.349)	2.943 (2.239, 3.865)
	**0.6**	**0.803 (0.611, 1.054)**	**1.124 (0.855, 1.476)**	1.445 (1.099, 1.897)	1.766 (1.344, 2.319)	2.087 (1.588, 2.740)	2.408 (1.832, 3.162)
	**0.8**	**0.679 (0.517, 0.892)**	**0.951 (0.723, 1.249)**	**1.222 (0.930, 1.605)**	1.494 (1.137, 1.962)	1.766 (1.344, 2.319)	2.037 (1.550, 2.676)
	**1.0**	**0.589 (0.448, 0.773)**	**0.824 (0.627, 1.082)**	**1.059 (0.806, 1.391)**	**1.295 (0.985, 1.700)**	1.530 (1.164, 2.010)	1.766 (1.344, 2.319)

eTable 3. Sensitivity analysis demonstrating the effect of a hypothetical unmeasured risk factor (confounder) on the adjusted odds ratio of the primary exposure of interest (hyperoxemia) on in-patient mortality at an adjusted odds ratio of the confounder of 4 and at varying prevalence in the exposed and unexposed groups. Values in cells represent the adjusted odds ratio of hyperoxia on in-patient mortality with 95% confidence intervals in parenthesis. Cells with bold text are those in which the 95% confidence interval for hyperoxemia crosses or is entirely below one.

		Prevalence in normoxemic group					
		**0.0**	**0.2**	**0.4**	**0.6**	**0.8**	**1.0**
Prevalence in hyperoxemic group	**0.0**	1.766 (1.344, 2.319)	2.825 (2.150, 3.710)	3.884 (2.956, 5.101)	4.944 (3.762, 6.493)	6.003 (4.568, 7.884)	7.063 (5.374, 9.275)
	**0.2**	**1.104 (0.804, 1.449)**	1.766 (1.344, 2.319)	2.428 (1.847, 3.188)	3.090 (2.351, 4.058)	3.752 (2.855, 4.928)	4.414 (3.359, 5.797)
	**0.4**	**0.803 (0.611, 1.045)**	**1.284 (0.977, 1.686)**	1.766 (1.344, 2.319)	2.247 (1.710, 2.951)	2.729 (2.076, 3.584)	3.210 (2.443, 4.216)
	**0.6**	**0.531 (0.480, 0.828)**	**1.009 (0.768, 1.325)**	1.387 (1.056, 1.822)	1.766 (1.344, 2.319)	2.144 (1.632, 2.816)	2.522 (1.919, 3.313)
	**0.8**	**0.519 (0.395, 0.682)**	**0.831 (0.632, 1.091)**	**1.142 (0.869, 1.500)**	1.454 (1.107, 1.910)	1.766 (1.344, 2.319)	2.077 (1.581, 2.728)
	**1.0**	**0.441 (0.336, 0.580)**	**0.706 (0.537, 0.928)**	**0.971 (0.739, 1.275)**	**1.236 (0.941, 1.623)**	1.501 (1.142, 1.971)	1.766 (1.344, 2.319)

© 2019 Ramgopal S et al. *JAMA Network Open.*

eTable 4. Sensitivity analysis demonstrating the effect of a hypothetical unmeasured risk factor (confounder) on the adjusted odds ratio of the primary exposure of interest (hyperoxemia) on in-patient mortality at an adjusted odds ratio of the confounder of 5 and at varying prevalence in the exposed and unexposed groups. Values in cells represent the adjusted odds ratio of hyperoxia on in-patient mortality with 95% confidence intervals in parenthesis. Cells with bold text are those in which the 95% confidence interval for hyperoxemia crosses or is entirely below one.

	Prevalence in normoxemic group						
Prevalence in hyperoxemic group		**0.0**	**0.2**	**0.4**	**0.6**	**0.8**	**1.0**
	**0.0**	1.766 (1.344, 2.319)	3.178 (2.418, 4.174)	4.591 (3.493, 6.029)	6.003 (4.568, 7.884)	7.416 (5.643, 9.739)	8.828 (6.718,11.594)
	**0.2**	**0.981 (0.746, 1.288)**	1.766 (1.344, 2.319)	2.550 (1.941, 3.349)	3.335 (2.538, 4.380)	4.120 (3.135, 5.411)	4.905 (3.732, 6.441)
	**0.4**	**0.679 (0.517, 0.892)**	**1.222 (0.930, 1.605)**	1.766 (1.344, 2.319)	2.309 (1.757, 3.032)	2.852 (2.170, 3.746)	3.396 (2.584, 4.459)
	**0.6**	**0.519 (0.395, 0.682)**	**0.935 (0.711, 1.228)**	**1.350 (1.027, 1.773)**	1.429 (1.088, 1.877)	2.181 (1.660, 2.864)	2.597 (1.976, 3.410)
	**0.8**	**0.420 (0.320, 0.552)**	**0.757 (0.576, 0.994)**	**1.093 (0.832, 1.435)**	**1.201 (0.914, 1.577)**	1.766 (1.344, 2.319)	2.102 (1.600, 2.761)
	**1.0**	**0.353 (0.269, 0.464)**	**0.636 (0.484, 0.835)**	**0.918 (0.699, 1.206)**	**1.201 (0.914, 1.577)**	1.483 (1.129, 1.948)	1.766 (1.344, 2.319)

© 2019 Ramgopal S et al. *JAMA Network Open.*

eTable 5. Descriptive data for encounters categorized by number of PaO_2 values corresponding with hyperoxia among patients with ≥3 PaO_2 measurements collected ≥3 hours apart.

	**No hyperoxemia**	**One hyperoxemic P_aO_2 value**	**Two hyperoxemic P_aO_2 values**	**Three or more hyperoxemic P_aO_2 values**
Number (%)	2,211 (63.8)	816 (23.6)	236 (6.8)	201 (5.8)
Median P_aO_2, mm Hg (IQR)	95 (75-128)	107 (79-146)	111 (79-156)	133 (87-206)
In-hospital mortality (%)	127 (5.7)	102 (12.5)	53 (22.5)	75 (37.3)
Number male (%)	1,229 (55.6)	481 (58.9)	133 (56.4)	115 (57.2)
Age				
<1 month	33 (1.5)	25 (3.1)	4 (1.7)	6 (3.0)
1 month to 1 year	337 (15.2)	143 (17.5)	47 (19.9)	38 (18.9)
1 year to 2 years	245 (11.1)	85 (10.4)	29 (12.3)	15 (7.5)
2 to 6 years	445 (20.1)	160 (19.6)	37 (15.7)	38 (18.9)
6 to 12 years	445 (20.1)	155 (19.0)	34 (14.4)	42 (20.9)
12 to 18 years	506 (22.9)	177 (21.7)	65 (27.5)	52 (25.9)
≥ 18 years	200 (9.0)	71 (8.7)	20 (8.5)	10 (5.0)
Race				
White	1,725 (78.0)	627 (76.8)	175 (74.2)	149 (74.1)
Black	319 (14.4)	120 (14.7)	42 (17.8)	25 (12.4)
Other/Not stated	167 (7.6)	69 (8.5)	19 (8.1)	27 (13.4)
Use of ECLS	36 (1.6)	35 (4.3)	16 (6.8)	41 (20.4)
Median Predicted % Mortality by m-PELOD 2 Score (IQR)	1.6 (0.7-4.7)	2.6 (1.0-9.4)	5.4 (1.2-22.4)	16.6 (3.25-45.2)

ECLS, extracorporeal life support; m-PELOD 2, modified Pediatric logistic organ dysfunction 2 score

© 2019 Ramgopal S et al. *JAMA Network Open.*

eTable 6. Association of the maximum PaO_2 during hospitalization and in-hospital mortality before and after adjustment with the m-PELOD 2 score, retaining only the last encounter for each patient during inclusion period (n=4,432 subjects).

	**Univariable analysis**		**Multivariable analysis**	
**Variable**	**OR (95% CI)**	**P**	**aOR (95% CI)**	**P**
Maximum P_aO_2				
No Hyperoxemia (<300mm Hg)	Ref	--	Ref	--
Hyperoxemia (≥300 mm Hg)	4.38 (3.54-5.42)	<0.001	1.55 (1.15-2.11)	0.005
m-PELOD-2 score	3.05 (2.80-3.33)	<0.001	2.91 (2.66-3.19)	<0.001
Received ECLS	12.76 (8.50-19.10)	<0.001	1.36 (0.78-2.38)	0.280
Number of ABGs during encounter	1.01 (1.01-1.01)	<0.001	1.00 (1.00-1.00)	0.500
Age (days)	1.00 (1.00-1.00)	0.759	--	--

Adjusted odds ratios (aOR) included variables of duration of hyperoxemia, modified pediatric logistic organ dysfunction (m-PELOD-2) score, provision of extracorporeal life support (ECLS), and number of arterial blood gases (ABG) per encounter. OR, odds ratio; CI, confidence interval

© 2019 Ramgopal S et al. *JAMA Network Open.*

eTable 7. Association of the number of PaO_2 measurements with hyperoxemia and in-hospital mortality, retaining only the last encounter for each patient during inclusion period (n=4,432 subjects). From these, patients with at least three P_aO_2 measurements at least three hours apart were analyzed (n=2,432).

	**Univariable analysis**		**Multivariable analysis**	
**Variable**	**OR (95% CI)**	**P**	**aOR (95% CI)**	**P**
Number of hyperoxemic P_aO_2				
No hyperoxemia	Ref	--	Ref	--
One P_aO_2 value ≥300 mmHg	2.21 (1.66-2.92)	<0.001	1.38 (0.94-2.03)	0.096
Two P_aO_2 values ≥300 mmHg	4.67 (3.22-6.76)	<0.001	1.67 (0.99-2.81)	0.054
Three or greater P_aO_2 values ≥300 mmHg	9.47 (6.60-13.59)	<0.001	2.60 (1.57-4.33)	<0.001
m-PELOD 2 score	3.00 (2.72-3.32)	<0.001	2.82 (2.54-3.13)	<0.001
Received ECLS	8.43 (5.49-12.93)	<0.001	1.56 (0.87-2.81)	0.139
Number of ABGs during encounter	1.01 (1.01-1.01)	<0.001	1.00 (1.00-1.00)	0.354
Age (days)	1.00 (1.00-1.00)	0.304	--	--

Adjusted odds ratios (aOR) included variables of number of hyperoxemic PaO2, modified pediatric logistic organ dysfunction (m-PELOD-2) score, provision of extracorporeal life support (ECLS), and number of arterial blood gases (ABG) per encounter. OR, odds ratio; CI, confidence interval

© 2019 Ramgopal S et al. *JAMA Network Open.*

eTable 8. Association of the maximum PaO_2 during hospitalization and in-hospital mortality using generalized estimating equations clustered by patient identifier.

	**Standard Error**	**Multivariable analysis**	
**Variable**		**aOR (95% CI)**	**P**
Maximum P_aO_2			
No Hyperoxemia (<300mm Hg)	--	Ref	--
Hyperoxemia (≥300 mm Hg)	0.141	1.75 (1.32-2.30)	<0.001
m-PELOD-2 score	0.040	2.60 (2.41-2.82)	<0.001
Received ECLS	0.282	1.36 (0.78-2.36)	0.276
Number of ABGs during encounter	0.001	1.00 (1.00-1.00)	0.851

Adjusted odds ratios (aOR) included variables of number of hyperoxemic PaO2, modified pediatric logistic organ dysfunction (m-PELOD-2) score, provision of extracorporeal life support (ECLS), and number of arterial blood gases (ABG) per encounter. CI, confidence interval

© 2019 Ramgopal S et al. *JAMA Network Open.*

eTable 9. Association of the number of PaO_2 measurements with hyperoxemia and in-hospital mortality among those patients with at least three PaO_2 measurements at least three hours apart as a sensitivity analysis using generalized estimating equations to account for clustering around subjects.

	**Standard Error**	**Multivariable analysis**	
**Variable**		**aOR (95% CI)**	**P**
Number of hyperoxemic P_aO_2			
No hyperoxemia	--	Ref	--
One P_aO_2 value ≥300 mmHg	0.176	1.48 (1.05-2.09)	0.027
Two P_aO_2 values ≥300 mmHg	0.224	2.01 (1.30-3.12)	0.002
Three or greater P_aO_2 values ≥300 mmHg	0.234	2.53 (1.60-4.00)	<0.001
m-PELOD 2 score	0.047	2.53 (2.31-2.77)	<0.001
Received ECLS	0.298	1.40 (0.78-2.50)	0.262
Number of ABGs during encounter	0.001	1.00 (1.00-1.00)	0.879

Adjusted odds ratios (aOR) included variables of duration of hyperoxemia, modified pediatric logistic organ dysfunction (m-PELOD-2) score, provision of extracorporeal life support (ECLS), and number of arterial blood gases (ABG) per encounter. CI, confidence interval

© 2019 Ramgopal S et al. *JAMA Network Open.*

eTable 10. Optimal thresholds derived from receiver operator curve analysis.

Criterion	Threshold	Sensitivity	Specificity	PPV	NPV	LR(+)	LR(-)
Youden	302	0.60 (0.55, 0.65)	0.76 (0.74, 0.77)	0.15 (0.14, 0.17)	0.96 (0.96, 0.97)	2.46 (2.25, 2.70)	0.53 (0.47, 0.60)
Misclassification Cost Term	641	0.03 (0.01, 0.05)	1.00 (1.00, 1.00)	0.65 (0.46, 0.77)	0.94 (0.88, 0.98)	26.46 (9.84, 71.18)	0.97 (0.96, 0.99)
Maximized specificity	714	0.00 (0.00, 0.02)	1.00 (0.99, Na)	1.00 (0.35, 1.00)	0.94 (0.64, Na)	n/a	9.95 (0.99, 1.00)
Maximized sensitivity and specificity	267	0.67 (0.62, 0.71)	0.66 (0.65, 0.68)	0.12 (0.12, 0.15)	0.97 (0.96, 0.97)	1.99 (1.84, 2.15)	0.50 (0.44, 0.58)
Maximum efficiency	641	0.03 (0.01, 0.05)	1.00 (1.00, 1.00)	0.65 (0.46, 0.77)	0.94 (0.88, 0.98)	26.46 (9.84, 71.18)	0.97 (0.96, 0.99)

Numbers in parenthesis represent 95% confidence intervals. PPV, positive predictive value; NPV, negative predictive value; LR(+), positive likelihood ratio; LR(-) negative likelihood ratio.

© 2019 Ramgopal S et al. *JAMA Network Open.*

eTable 11. Multivariable logistic regression model examining additional thresholds of maximum PaO_2 values

	Univariable analysis		Multivariable analysis	
**Variable**	**OR (95% CI)**	**P**	**aOR (95% CI)**	**P**
Maximum P_aO_2				
No Hyperoxemia (<300mm Hg)	Ref	--	Ref	--
Hyperoxemia (300-499 mm Hg)	3.82 (3.05-4.79)	<0.001	1.56 (1.16-2.09)	0.003
Extreme hyperoxemia (≥500 mm Hg)	7.77 (5.77-10.47)	<0.001	2.38 (1.60-3.53)	<0.001
m-PELOD-2 score	2.71 (2.53-2.91)	<0.001	2.59 (2.40-2.80)	<0.001
Received ECLS	10.68 (7.46-15.30)	<0.001	1.33 (0.82-2.16)	0.253
Number of ABGs during encounter	1.01 (1.01-1.01)	<0.001	1.00 (1.00-1.00)	0.902
Age (days)	1.00 (1.00-1.00)	0.362	--	--

Adjusted odds ratios (aOR) included variables of duration of hyperoxemia, modified pediatric logistic organ dysfunction (m-PELOD-2) score, provision of extracorporeal life support (ECLS), and number of arterial blood gases (ABG) per encounter. OR, odds ratio; CI, confidence interval

eTable 12. Proportions of PaO_2 corresponding with hyperoxia at different fractions of inspired oxygen (FiO_2) when oxygen saturation (SpO_2) was 100% from all encounters in which an ABG was done within 20 minutes of a charted FiO_2 and SpO_2.

**FiO2**	0.60-0.69	0.70-0.79	0.80-0.89	0.90-0.99	1.00
**SpO2**	100%	100%	100%	100%	100%
**Number of events**	1,244	600	458	395	907
**ABG with hyperoxia (≥300mm Hg) (percent)**	31 (2.5)	50 (8.3)	74 (16.2)	80 (20.3)	234 (25.8)
**Median P_aO_2 (IQR)**	141 (105-187)	146 (109-212)	170 (113-243)	182 (124-266)	195 (126-304)
**Medan P_aO_2 (±SD)**	153 (63)	168 (79)	194 (102)	212 (121)	229 (135)

© 2019 Ramgopal S et al. *JAMA Network Open.*
